# Transcriptome instability in colorectal cancer identified by exon microarray analyses: Associations with splicing factor expression levels and patient survival

**DOI:** 10.1186/gm248

**Published:** 2011-05-27

**Authors:** Anita Sveen, Trude H Ågesen, Arild Nesbakken, Torleiv O Rognum, Ragnhild A Lothe, Rolf I Skotheim

**Affiliations:** 1 Department of Cancer Prevention, Institute for Cancer Research, The Norwegian Radium Hospital, Oslo University Hospital, PO Box 4953 Nydalen, NO-0424 Oslo, Norway; 2 Centre for Cancer Biomedicine, Faculty of Medicine, University of Oslo, PO Box 1072 Blindern, NO-0316 Oslo, Norway; 3 Department of Gastrointestinal Surgery, Aker University Hospital, Oslo University Hospital, PO Box 4959 Nydalen, NO-0424 Oslo, Norway; 4 Institute of Forensic Medicine, University of Oslo, PO Box 1072 Blindern, NO-0316 Oslo, Norway

## Abstract

**Background:**

Colorectal cancer (CRC) is a heterogeneous disease that, on the molecular level, can be characterized by inherent genomic instabilities; chromosome instability and microsatellite instability. In the present study we analyze genome-wide disruption of pre-mRNA splicing, and propose transcriptome instability as a characteristic that is analogous to genomic instability on the transcriptome level.

**Methods:**

Exon microarray profiles from two independent series including a total of 160 CRCs were investigated for their relative amounts of exon usage differences. Each exon in each sample was assigned an alternative splicing score calculated by the FIRMA algorithm. Amounts of deviating exon usage per sample were derived from exons with extreme splicing scores.

**Results:**

There was great heterogeneity within both series in terms of sample-wise amounts of deviating exon usage. This was strongly associated with the expression levels of approximately half of 280 splicing factors (54% and 48% of splicing factors were significantly correlated to deviating exon usage amounts in the two series). Samples with high or low amounts of deviating exon usage, associated with overall transcriptome instability, were almost completely separated into their respective groups by hierarchical clustering analysis of splicing factor expression levels in both sample series. Samples showing a preferential tendency towards deviating exon skipping or inclusion were associated with skewed transcriptome instability. There were significant associations between transcriptome instability and reduced patient survival in both sample series. In the test series, patients with skewed transcriptome instability showed the strongest prognostic association (*P *= 0.001), while a combination of the two characteristics showed the strongest association with poor survival in the validation series (*P *= 0.03).

**Conclusions:**

We have described transcriptome instability as a characteristic of CRC. This transcriptome instability has associations with splicing factor expression levels and poor patient survival.

## Background

Colorectal cancer (CRC) is a prevalent disease with a world-wide incidence of more than one million new cases each year, making it the third most commonly diagnosed cancer among men and women [1]. Colorectal tumors are heterogeneous and evolve through multiple pathways. Malignant transformation is dependent on the accumulation of numerous genetic changes over years. Such genetic instability provides a way of classifying tumors into different molecular subtypes [2]. CRCs with the microsatellite instability (MSI) phenotype have a defective mismatch repair system, which results in a high proportion of mutations in nucleotide repeats (microsatellites) throughout the genome. Mismatch mutations of microsatellites located in coding-regions may exert tumorigenic effects - for example, by inactivating tumor suppressor genes [3,4]. Cancers with MSI generally have few numeric changes or rearrangements at the chromosomal level. In contrast, CRCs with chromosomal instability (CIN) exhibit frequent chromosomal rearrangements and aneuploidy [5]. MSI and CIN tumors represent two different types of virtually mutually exclusive genetic instabilities, and also two separate mechanistic mutational pathways for CRC development [2]. A third subgroup, comprising both MSI and CIN tumors, is denoted as having a CpG island methylator phenotype (CIMP). Although these tumors often harbor *BRAF *mutations, CIMP is considered an epigenetically rather than genetically acting phenotype, and is characterized by extensive DNA methylation at promoter regions and associated gene silencing [6,7]. Despite the fact that the above described refinements to CRC classification take into account molecular characteristics, it is evident that the total genetic definition of this heterogeneous disease is yet to be described [6].

Tumor staging remains the most important factor for determining the prognosis of CRC patients [8]. Colorectal tumors are divided into four stages by the tumor-node-metastasis (TNM) system, taking into account depth of infiltration of the tumors, the extent of lymph node involvement, and the presence of distant metastases [9]. During the past few years, molecular markers with potential prognostic value have been identified, several with relationships to the genetic and epigenetic types of instabilities mentioned above [10]. However, no such markers are currently recommended for clinical use, and the need for discovery of novel biomarkers to aid the determination of prognosis in CRC patients remains great.

Alternative pre-mRNA splicing is an important source of functional diversity in the majority of mammalian genes [11]. Nearly all multi-exon genes are expressed in multiple isoforms [12]. Alternative splicing may occur as cassette alternative exons, mutually exclusive exons, intron retentions, or alternative 3' or 5' splice sites. Additionally, transcript variation may be caused by alternative promoter usage, resulting in alternative first exons, or alternative polyadenylation sites, presented as alternative terminal exons [13]. Three consensus sequence elements are required for splicing to occur, two at each border of the intron, as well as the intronic branch site. Additionally, splicing is regulated by other *cis*-acting elements (exonic and intronic splicing silencers and enhancers) as well as the numerous *trans*-acting factors constituting the splicing machinery [14,15]. The integrity of this wide range of elements is crucial for splicing fidelity and the maintenance of a stable and properly functioning transcriptome.

Aberrant splicing patterns have been associated with cancer [16]. One example is the shift in expression towards the anti-apoptotic isoform (*BCL-X*_*L*_) of the apoptosis regulator *BCL-X *[17]. Cancer-specific transcript variation has also been found in CRC [18,19]. Recently, it has been shown that global splicing patterns are likely to be distorted in several cancers [20], and preferential utilization of alternative splice sites is found to be a property of tumors [21,22]. Moreover, differential expression of splicing factors is reported in cancer tissues compared to normal tissue [23]. For some cancers - for example, CRC - this is, for the most part, seen as up-regulated expression [24]. Despite previous publications on general changes in expression levels of splicing factors in cancer, it is yet to be determined what implications this imposes on the cancer phenotype.

In this study, we describe the disruption of alternative splicing as a global event in CRC tissue using exon microarray analysis. We show a great extent of variation in the amount of deviating exon usage among the samples. This transcriptome instability is found to be associated with prognosis in CRC patients, and also has clear associations with the expression levels of approximately half of all splicing factors.

## Methods

### Material

Two independent series comprising a total of 160 stage II and III CRC tissue samples were included in this study. The two series are referred to as test and validation series (Table 1). The test series consisted of 83 stage II and III CRC tissue samples collected from patients treated surgically for CRC in hospitals in the Oslo-region from 1987 to 1989. To ensure adequate group sizes for survival analyses, the patients were selected to have a 10-year overall survival of approximately 50%, as well as approximately equal amounts of recurrences within the two stages. The MSI status of the tumors had previously been determined [25]. The independent validation series of 77 stage II and III CRC tissue samples were consecutively collected from patients undergoing complete resection at Aker University Hospital, Oslo, in the period 2005 to 2007. These patients were subjected to the current treatment regime, with routine administration of postoperative chemotherapy in an adjuvant setting when presenting with stage III tumors. The patients received no radiation therapy prior to surgery. MSI status of the tumors in the validation series was determined in the same manner as for the test series. Additionally, normal colonic mucosa taken from disease-free areas distant to the primary tumors of each of 13 patients in the validation series was included in the analysis (six and seven stage II and III tumors, respectively, and six tumors with MSI). The research conformed to the Helsinki Declaration and the research biobanks have been registered according to national legislation (numbers 2781 and 236-2005-16141). The study (amendment number 2010/1805) is part of a project approved by the Regional Committee for Medical and Health Research Ethics (numbers 1.2005.1629 and S-09282c 2009/4958), which requires that informed consent is obtained from patients being enrolled to the study. RNA was extracted from the CRC samples using the Qiagen AllPrep DNA/RNA Mini Kit (Qiagen GmbH, Hilden, Germany), and the Ambion RiboPure™ kit (Life Technologies, Carlsbad, CA, USA) was used for the normal colonic mucosa samples. Both procedures were performed according to the manufacturers' protocols.

**Table 1:** Clinicopathological and molecular characteristics of the two independent colorectal cancer study populations

	Test series (*n *= 83)	Validation series (*n *= 77)
Age at diagnosis (mean ± SD)	66.0 ± 11.7	72.7 ± 13.5
Sex (male; female)	40; 43	33; 44
Stage (II; III)	46; 37	44; 33
Location (right; left; rectum)	26; 25; 32	46; 20; 11
Mean follow-up, years (minimum; maximum)	6.7 (0.7; 10.0)	3.5 (0.2; 5.0)
Number of events (deaths from CRC)	41	10
MSI	13	24
sTIN^a^	12 (15%)	24 (31%)
oTIN^b^	14 (17%)	30 (39%)
Either sTIN, oTIN, or both	24 (29%)	43 (56%)
Both TIN phenotypes	2 (2%)	11 (14%)

^a^sTIN, skewed transcriptome instability; preferential exon inclusion or skipping (difference in relative amounts of aberrant exon skipping and inclusion greater than ± 0.7). ^b^oTIN, overall transcriptome instability; total relative amounts of aberrant splicing greater than ± 1.0. SD, standard deviation; TIN, transcriptome instability.

### Exon microarray analysis

RNA (1 μg) from each sample was individually amplified, reverse transcribed, fragmented, and labeled using the Affymetrix GeneChip^® ^Whole Transcript (WT) Sense Target Labeling Assay [26]. Labeled sense strand DNA was hybridized onto the Affymetrix GeneChip Human Exon 1.0 ST Array for 16 to 18 hours [27]. Each array contains 1.4 million probe sets, of which 289,961 target well annotated full-length human mRNAs ('core' probe sets), and the remaining probe sets are derived from annotations of lower confidence levels, as well as computer predictions [28]. A probe set corresponds approximately to one exon, and will be referred to as such herein. The arrays were finally washed, stained and scanned according to the manufacturer's protocol.

### Data analysis

Scanning of the microarrays and preprocessing of raw image intensity data were controlled by the Affymetrix GeneChip Command Console software (version 1.0). For each microarray, the software generated cell intensity (CEL) files storing probe-level intensity data calculated from scanned image files containing pixel intensity values. CEL data files were used as input for preprocessing and alternative splicing detection with the Finding Isoforms using Robust Multichip Analysis (FIRMA) method [29] (Additional file 1). As part of the FIRMA method, the first two preprocessing steps of the microarrays were performed according to the robust multi-array average (RMA) approach, involving background correction of perfect match probes and inter-chip quantile normalization [30]. The summarization step estimating gene expression levels was slightly modified from standard RMA, not taking into account the chip-exon effect, that is, ruling out the relative change for the sample in a particular exon. For this purpose, a custom made chip definition file containing 284,258 probe sets targeting exons belonging to the 'core' set of well annotated exons was downloaded from aroma.affymetrix [31]. Applying this annotation file, the collective set of exons made up 18,708 transcript clusters, or genes. Alternative splicing scores, FIRMA scores, were calculated for each individual exon in each individual sample to represent a measure for whether differential exon usage has occurred. These scores were calculated as exon-level intensities deviating from the corresponding gene level, assessed indirectly as the residual after fitting the gene-level model to the actual data. Large residuals indicated differential expression of the particular exon compared to the corresponding gene level [29]. The FIRMA scores were log-2 transformed. The microarray data can be accessed from NCBI's Gene Expression Omnibus (GEO) with the accession number [GEO:GSE24551].

To provide a global estimate of the relative amount of differential exon usage per sample, we counted the sample-wise numbers of probe set level FIRMA scores belonging to the upper and lower 1st percentiles of all FIRMA scores in the data sets.

For further statistical analyses, the software SPSS 15.0 (SPSS Inc., Chicago, IL, USA) was used. This includes t-statistics, multinomial logistic regression, generation of Kaplan-Meier plots, Cox regression analyses for calculation of hazard ratios (HR) and corresponding 95% confidence intervals (CI), Fisher's exact test, and Mantel-Cox test for equality of survival distributions. *P*-values < 0.05 were considered significant. Hierarchical clustering analysis was done using J-Express 2011 (MolMine AS, Bergen, Norway).

### Splicing factors

A list of 280 human splicing factors (Table S1 in Additional file 2) was created by combining results from the Gene Ontology project [32] and Swiss-Prot at the UniProt Knowledgebase [33] in July 2009. Using the AmiGO web application [34], the Gene Ontology database was searched for the terms 'nuclear mRNA splicing, via spliceosome' (GO:0000398) and 'spliceosomal complex' (GO:0005681). The ExPASy proteomics server [35] was used to search Swiss-Prot for human proteins with the terms 'splicing' and 'spliceosome'. Thirty-one additional genes were added to the list based on their splicing-related descriptions, as found using the GeneCards Human Gene Database [36]. Gene level expression data for these splicing factor genes were independently obtained from the CEL files of the CRC samples in the two series. The expression data were summarized on background-corrected and quantile-normalized data using the RMA algorithm implemented in the Affymetrix Expression Console 1.1 software.

For comparison, 100 gene sets with 280 genes each were created by random sampling using the R statistical software (Additional file 1). Expression levels for these genes were obtained from the test series of CRC samples, in the same manner as for the splicing factor genes.

## Results

### Variation in the amounts of aberrant alternative exon usage among colorectal cancer tissue samples

**Figure 1:**
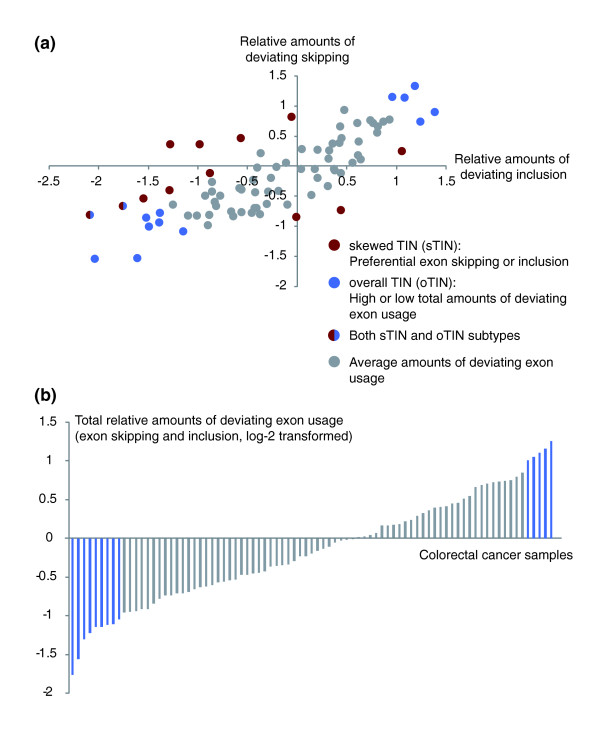
**Distribution of relative amounts of deviating exon usage in the CRC test series**. The axes represent the log-2 amounts of deviating exon usage relative to the average amount per sample. **(a) **Sample-wise comparison of deviating exon skipping and inclusion events for the 83 CRCs in the test series. **(b) **A combination of exon skipping and inclusion events constitutes the total relative amounts of deviating exon usage. Blue bars mark samples with the overall transcriptome instability (oTIN) subtype. TIN, transcriptome instability.

Exon microarray profiles from a test series of 83 CRC tissue samples were investigated for global differences in alternative exon usage. To indicate to what extent the expression level of an exon deviated from the overall expression level of the gene in which it is encoded, we calculated an alternative splicing score based on the FIRMA algorithm [29]. A total of 284,258 exons were scored in each of the 83 CRC samples. The log-2 transformed scores followed a normal distribution (Figure S1 in Additional file 2). Strong negative and positive scores are indications of, respectively, alternative exon skipping (exclusion) and inclusion that deviate from the general pattern among the cancer samples. The lower and upper 1st percentiles across all samples were -2.2 and 1.9, and these values were used as thresholds for scoring deviating exon skipping and inclusion. For each sample in the test series, a count was made of the number of exons with values exceeding these thresholds. The average combined number of deviating exon skipping and inclusion per sample was 5,685 (range 1,666 to 13,638). The average amount of exon skipping was 2,843 (range 974 to 7,171), the same as for exon inclusion (range 668 to 7,437). In the following, we report the sample-wise log-2 transformed amounts of deviating exon usage relative to average values in the dataset. These values are referred to as relative amounts of deviating skipping, inclusion or exon usage, the latter representing the combination of the two former, that is, the total sample-wise amounts of differential exon usage (Figure 1).

We consider tumors with large amounts of deviating exon usage as tumors with transcriptome instability (TIN). Tumors showing preferential exon skipping or inclusion - that is, having a skewed distribution in the amounts of deviating skipping and inclusion events (difference in relative amounts of deviating skipping and inclusion greater than ± 0.7; *n *= 12) - are considered to have a skewed TIN (sTIN) subtype. Tumors where the overall amounts of deviating exon usage differ from the average (total relative amounts of deviating exon usage greater than ± 1.0; *n *= 14) are considered to have an overall TIN (oTIN) subtype. The patients showed no significant associations between sTIN or oTIN and MSI status, tumor stage, patient age and gender, or tumor location (by multinomial logistic regression).

To investigate whether this large sample-to-sample variation in deviating exon usage amounts was cancer specific, comparisons were made between 13 paired CRC and normal colonic mucosa samples from the validation series. This comparison was conducted in a manner that explored differential exon usage in each sample pair individually, normalized against the background level of differential exon usage occurring in normal colonic mucosa (Additional file 1). The total relative amounts of deviating exon usage were significantly higher in the cancer samples compared to their normal counterparts (*P *= 0.003 by paired samples *t*-test; Figure S2A in Additional file 2). To ensure that the scores given by the FIRMA algorithm truly are sensitive to differential exon usage between CRC and normal colonic mucosa, we investigated the alternative splicing scores of splicing events that have previously been predicted and validated in exon microarray analyses [18,19]. Of 13 exons known to be alternatively spliced between CRC and normal colonic mucosa (indicated with exon array probe set IDs in Table S2 in Additional file 2), 11 showed a mean difference in alternative splicing scores between the paired samples according to expectation (Figure S2B in Additional file 2). Moreover, across the 13 CRC samples, 36% of the probe sets targeting these alternatively spliced exons were assigned a score exceeding the upper or lower 1st percentile thresholds, and have accordingly been accounted for in the total relative amounts of deviating exon usage in CRC compared to normal colonic mucosa.

### Correlation between oTIN and expression levels of splicing factors

**Figure 2:**
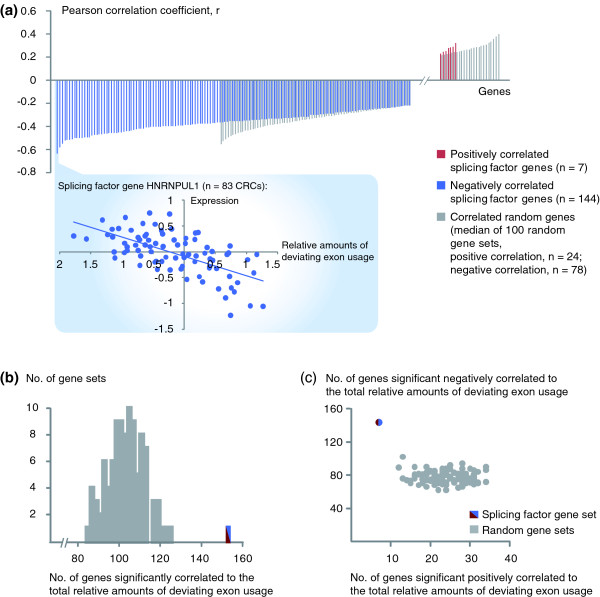
**Correlation between the oTIN subtype and expression levels of splicing factors**. (**a) **Pearson correlation coefficients (r) for the 151 splicing factors with expression levels significantly correlated to the total relative amounts of deviating exon usage (*P *< 0.05). Shown with grey bars are significantly correlated genes representing the median from 100 randomly selected gene sets of equal size (*n *= 102). Plotted below is the expression level versus deviating exon usage amounts per sample for the splicing factor gene with the strongest correlation (*HNRNPUL1*, r = -0.63, both axes are median-centered and log-2 transformed). **(b) **The splicing factor gene set has more genes (*n *= 151) significantly correlated to the total relative amounts of deviating exon usage than each of 100 random gene sets (range 84 to 124). **(c) **The splicing factor gene set has more genes with significant negative correlation to deviating exon usage amounts, and fewer genes with significant positive correlation, compared to 100 random gene sets.

Within the test series, the expression levels of 54% of splicing factors (151 of 280) showed a significant correlation to the total relative amounts of deviating exon usage (Pearson correlation, *P *< 0.05; Figure 2A). To test whether this correlation is stronger than expected by chance, 100 random sets of 280 genes were constructed and analyzed for correlation to sample-wise deviating exon usage amounts in the same manner (Additional file 1). The amounts of significantly correlated genes among these random gene sets were significantly lower than for the splicing factor gene set (range 30 to 44%; *P *< 0.01; Figure 2B). Also, considering only genes with significant correlation to deviating exon usage amounts, the mean r among the splicing factor genes was -0.33. This was significantly stronger in the direction of negative correlation compared to the random gene sets (*P *< 0.0001 by independent samples *t*-test for equality of means; Table S3 in Additional file 2). Notably, the majority of significantly correlated splicing factor genes (144 of 151) was negatively correlated to the amounts of deviating exon usage, that is, 21 times more than the amount of positively correlated splicing factor genes. This ratio was significantly higher than the corresponding ratios among the 100 individual random gene sets (range 2.1 to 7.8; *P *< 0.01; Figure 2C). To further explore the significance of these strong correlations, the corresponding correlations were calculated for 1,000 permutations of the amounts of deviating exon usage across the samples (Additional file 1). The median Pearson correlation coefficient for all splicing factor genes (*n *= 280) in each permutation ranged from -0.17 to 0.18 (Figure S3A in Additional file 2), all weaker than for the observed amounts of deviating exon usage (r = -0.23, hence *P *< 0.001). Also, the increase in amounts of negatively compared to positively correlated splicing factor genes was higher for the observed deviating exon usage amounts than for 99% of the permutations (Figure S3B in Additional file 2).

**Figure 3:**
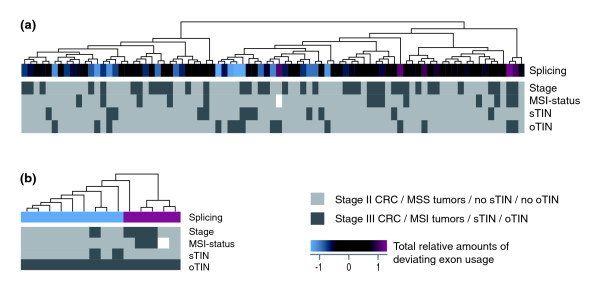
**Hierarchical clustering analyses of CRC test samples by expression levels of all splicing factors**. **(a) **Unsupervised hierarchical clustering analysis of all 83 CRC samples based on the expression levels of all 280 splicing factor genes separates the samples into clusters with predominantly lower (blue boxes) and higher (red boxes) relative amounts of deviating exon usage than the average sample (black boxes), according to the oTIN subtype. **(b) **Samples considered to have the oTIN subtype were almost completely separated into two groups with low and high relative amounts of deviating exon usage after hierarchical clustering based on the expression levels of the total set of splicing factors. Both clusters were created using Euclidean distance metrics and complete linkage. MSS, microsatellite stability.

To investigate whether the expression levels of splicing factors could separate CRC samples according to the oTIN subtype, samples in the test series were hierarchically clustered based on the expression levels of the total set of 280 splicing factor genes (Euclidean distance metrics, complete linkage). Groups of samples with high and low total relative amounts of deviating exon usage were mainly separated into different clusters (Figure 3A). Restricting the hierarchical clustering to the oTIN samples resulted in an almost complete separation into the two respective groups (Figure 3B). This sample clustering was independent of tumor stage and MSI status. Also, the cancers did not cluster based on the sTIN subtype.

### Associations between TIN and poor patient survival

**Figure 4:**
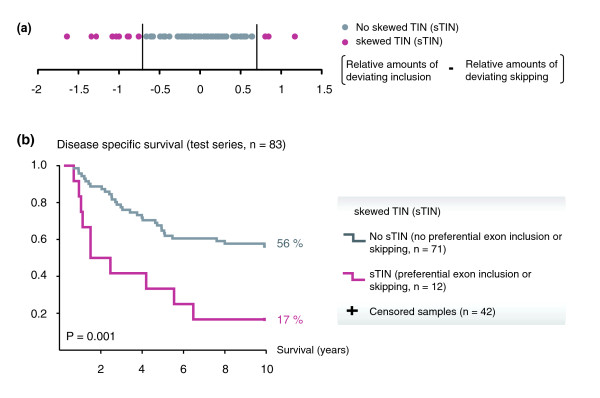
**Association between the sTIN subtype and patient survival in the CRC test series**. **(a) **Differences between the amounts of deviating exon inclusion and skipping per sample were used to identify patients with sTIN tumors (threshold at ± 0.7 on the log-ratio scale). **(b) **Disease-specific survival among patients in the test series stratified by the sTIN subtype. In this analysis, deaths from CRC were considered events (*n *= 41). Patients who survived throughout the 10 years of follow-up were censored (*n *= 42). Recurrences (*n *= 2 among patients who survived) were ignored.

Patients with sTIN tumors (Figure 4A) had significantly reduced survival compared to the patients not characterized with preferential exon inclusion or exclusion. The 10-year disease-specific survival rates were 17 and 56%, respectively (*P *= 0.001 by log rank test for equality of survival distributions; Figure 4B; HR = 3.2; 95% CI, 1.5 to 6.5). Either variant of sTIN, preferential exon inclusion or exclusion, was associated with poor patient survival (non-significant association for exon inclusion; Table S4 in Additional file 2). Similar results were found when applying different stratification thresholds for preferential exon inclusion and/or skipping (Table S4 in Additional file 2). Also when analyzing for disease-free survival, patients with sTIN cancers had a significantly reduced survival rate (HR = 2.9; 95% CI, 1.4 to 6.0; *P *= 0.002).

In a multivariate model including tumor stage, MSI status, patient age and gender, as well as tumor location, sTIN was the strongest independent prognostic predictor (HR = 3.5; 95% CI, 1.5 to 8.3; *P *= 0.004). Among the other variables, only tumor stage showed significant associations with patient prognosis in this model.

Patients whose tumors were characterized by oTIN had a slightly poorer survival rate than patients with average amounts of deviating exon usage, although this finding was not significant (Table S5A in Additional file 2). However, patients with either sTIN or oTIN cancers (*n *= 24) had significantly poorer survival than patients with TIN-negative cancers (HR = 2.1; 95% CI 1.1 to 3.9; *P *= 0.02). This difference was significant also in a multivariate model including tumor stage, MSI status, patient age and gender, as well as tumor location (HR = 3.2; 95% CI, 1.5 to 6.7; *P *= 0.002). Two patients had tumors with overlapping subtypes of TIN, that is, characterized by both sTIN and oTIN. These patients died from metastatic disease 0.8 and 4.3 years after surgical removal of their primary tumor.

### Validation of TIN in an independent series of stage II and III colorectal cancers

**Figure 5:**
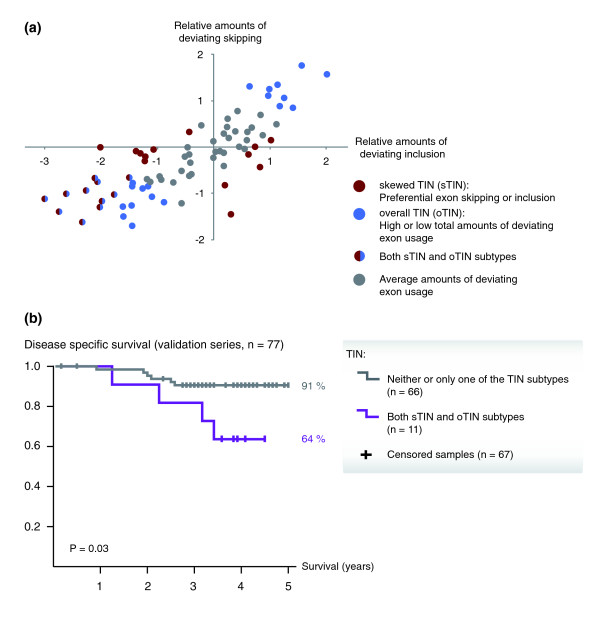
**Transcriptome instability in the colorectal cancer validation series**. **(a) **Sample-wise comparison of deviating exon skipping and inclusion events for the 77 CRCs in the validation series. **(b) **Patients in the validation series whose tumors where characterized with both sTIN and oTIN had a significantly lower 5-year disease-specific survival rate than patients whose tumors were characterized with none or only one of the two TIN subtypes. Deaths from CRC were considered events (*n *= 10). Censoring occurred at 5 years for survivors (*n *= 61), and at time of incidence for causes of death other than CRC (*n *= 6). Recurrences among survivors (*n *= 9) were ignored.

Transcriptome instability was tested also in an independent validation series of 77 stage II and III CRCs. Applying the same thresholds for characterizing tumors with TIN as in the test series, there were 30 samples in the validation series with oTIN, and 24 samples with sTIN (Additional file 2). Eleven of the samples had overlapping phenotypes, that is, assigned to both the sTIN and oTIN subtype groups (Figure 5A).

As in the test series, there were no significant associations between either of the two TIN subtypes and MSI status, tumor stage, patient age or gender, or tumor location (by multinomial logistic regression).

Also in the validation series, there was a strong association between oTIN and expression levels of splicing factors. Here, the sample-wise expression levels of 48% of splicing factors (133 of 280) were significantly correlated to the total relative amounts of deviating exon usage (compared to 54% in the test sample series; Pearson correlation, *P *< 0.05; Figure S4A in Additional file 2). Furthermore, the significant shift towards stronger negative correlation among splicing factor genes compared to random gene sets was also indicated in the validation series. In fact, 129 of 133 (97%) significantly correlated splicing factor genes were negatively correlated.

As in the test series, samples in the validation series were separated according to the oTIN subtype by hierarchical clustering analysis of expression levels of splicing factor genes (Euclidean distance metrics, complete linkage; Figure S4B in Additional file 2). Although the amount of oTIN samples was higher than in the test sample series (*n *= 30 compared to *n *= 14), the oTIN samples were almost completely separated into the two respective groups (Figure S4C in Additional file 2). Also in the validation series, the clustering was independent of tumor stage and MSI status.

The strong associations between TIN and disease-specific survival among the patients in the test series were not as clearly indicated in the validation series (Table S5B in Additional file 2). Notably, the mean follow-up period of the patients was considerably shorter (3.5 years) than in the test series (6.7 years). However, using the same stratification thresholds as in the test series, patients whose tumors were characterized with both of the TIN subtypes (*n *= 11) had a significantly poorer 5-year survival rate (64%) than patients whose tumors were characterized with neither or only one of the TIN subtypes (*n *= 66, 91% survival rate, *P *= 0.03; Figure 5B; HR = 3.7; 95% CI, 1.1 to 13.6). In a multivariate model including tumor stage, MSI status, patient age and gender, as well as tumor location, TIN was close to being an independent predictor of poor prognosis (multivariate HR = 3.1; 95% CI 0.9 to 11.2; *P *= 0.08).

## Discussion

In this study we have described TIN, characterized by deviating mRNA splice variant patterns, in CRC. Two main subtypes of this characteristic are described, oTIN and sTIN. The oTIN subtype was demonstrated through great variation in the total amounts of deviating exon usage among CRC tissue samples. This characteristic was found to be associated with the expression levels of approximately half of all splicing factors in two independent sample series. The sTIN subtype separated the samples by the preferred usage of alternative exon skipping or inclusion. TIN was shown to be associated with poor patient survival in two independent sample series, although prognostic stratification was achieved by different TIN subtypes in the two series. In the test series, sTIN was shown to have the strongest prognostic value, while a combination of the two subtypes was the most strongly associated with poor patient survival in the validation series.

Genomic instability is an important classifier of colorectal tumors because of its associations with certain risk factors and clinical features [6]. Such genomic instability includes the virtually non-redundant differentiation between CIN and MSI. More recently, a classifier of the epigenetic state of the genome, CIMP, has also been introduced as an important phenotype describing the molecular nature of CRC [37,38]. In analogy to these molecular classifiers of the CRC genome, TIN is here proposed as a potential classifier of the CRC transcriptome.

Transcriptome instability has potential clinical value. From our analyses it is not evident which of the two TIN subtypes is more closely related to patient survival, as the results differed between the test and validation series. It is not clear whether this may be due to the different clinicopathological constitution of the two patient series. Notably, the mean follow-up period of the patients was considerably shorter in the validation series (3.5 years, compared to 6.7 years in the test series). These patients received adjuvant chemotherapy according to the current standard, whereas patients in the historical test series received no other treatment than surgery. The mean age at diagnosis was also different between the two patient series (66 versus 73 years). These factors may have contributed to the observed different associations between TIN and patient survival. Although we found no associations between MSI and TIN, the different amounts of patients with MSI tumors in the two series (16% versus 31%) may also have influence in this regard. However, both series showed significantly reduced survival for patients stratified according to TIN, suggesting that high amounts of inter-tumor deviations in exon usage patterns may indeed be associated with poor patient survival. We would welcome additional effort to verify the prognostic value of this molecular characteristic.

The amounts of samples assigned to either or both of the TIN subtypes varied between the two sample series. In the test series, 29% (24 of 83) of the tumors were characterized by TIN, compared to 56% in the validation series (43 of 77). Although resulting in an increased amount of TIN-positive samples, we chose to keep a fixed scoring threshold for TIN to avoid introducing subjective bias into the validation. Despite the different frequencies, the reproducibility of the strong associations between oTIN and expression levels of splicing factor genes provides strong evidence for a biological explanation of the observed variability in deviating exon usage amounts. However, it is not certain to what extent this has influenced the associations between TIN and patient survival in the two series.

The amounts of deviating exon usage were significantly higher in the cancer samples compared to paired normal colonic mucosa. A possible bias may have been introduced to these comparisons by the use of different RNA extraction protocols for the two sample groups. However, when analyzing known splicing events, 11 of 13 exons were found to have a mean difference in alternative splicing scores between the paired samples according to expectation. Also, 36% of the probe sets across the 13 CRC samples included in this analysis of known splicing events were designated as differentially spliced (exceeding the upper and lower 1st percentiles of alternative splicing scores). Accordingly, it seems evident that alternative exon usage is indeed reflected in the analytical approach, where individual exons in individual samples are scored according to the likelihood of differential splicing. For the majority of the known splicing events tested, the recurrence rate in CRC tissue is uncertain, due to limited sample numbers used for discovery [18]. The cancer-specific splicing event of *SLC39A14*, however, is reported to have high cancer sensitivity [19]. In accordance with this, exon 4A in this gene was designated as differentially excluded in all CRC samples relative to normal mucosa (<1st percentile of alternative splicing scores). In contrast, in the inter-tumor comparisons that are the main focus here, the majority of the cancer samples showed no signs of differential splicing for this exon, reflecting the nature of the current study, investigating exon usage variation among tumor samples and not between tumor and normal samples. Since the amounts of aberrant splicing were found to be higher in the cancer samples than their normal counterparts, we find it likely that TIN is most relevant to studies of cancer tissues. It remains uncertain whether this is specific for CRC or may be a common characteristic for cancers in other tissues as well.

It is striking that the expression levels of the majority of splicing factors negatively correlated to the numbers of deviating exon usage. Irrespective of the role of the splicing factor, whether it is predominantly a splicing enhancer or silencer, and irrespective of the type of exon usage event (exon skipping or inclusion), this was true for more than 95% of the significantly correlated splicing factor genes in two independent sample series. In fact, the correlations among splicing factor genes were significantly stronger in the direction of negative correlation than among random gene sets of equal size. This strong correlation is further supported by unsupervised hierarchical clustering analyses, which were based on the expression levels of the total set of splicing factors and separated the samples according to the oTIN phenotype in both series. Therefore, the association between low expression levels of splicing factors and increased variability in exon usage seems to be indicative of a critical role of splicing factor activity for the maintenance of a stable transcriptome. This suggests a biological rationale for the differences in exon usage observed in this study.

Consistent with this great variation in exon usage among CRC samples, other recent studies have also shown that the extent of alternative splicing far exceeds previous estimates. On average, individual multi-exon genes are suggested to undergo at least seven alternative splicing events across various human tissues and cell lines [12,39]. This provides the possibility for tremendous variation in transcriptome composition. Several studies have reported genome-wide distortion of alternative splicing in cancer tissues [21,22]. It has also been shown that alternative splicing in cancer exhibits both tissue dependency and dependency upon the type of splicing event considered [21]. Intron retention and cassette alternative exons have been suggested to be more prevalent in normal tissues, and alternative 3' and 5' splice sites to occur more often in cancers. Various splicing events are also believed to occur at different levels in different cancer types. This indicates a complex distortion of exon splicing in cancer. Due to the composition of the exon microarrays and the nature of the splicing detection algorithm, the current study describes primarily the alternative skipping and inclusion of individual exons. In terms of splicing events, this essentially represents intron retention and cassette alternative exons. Patterns of mutual exclusion among exons are not considered. Transcript variation not attributed to alternative splicing, but rather to alternative promoter usage or polyadenylation sites, is also represented in the analysis, as all flanking exons are treated similarly to exons contained internally within transcripts. A detailed analysis of alternative usage of promoters and polyadenylation sites requires a detailed description of transcript structure and abundance, which is beyond the scope of this study.

Although the approach taken here to analyze sample-wise amounts of deviating exon usage was shown to correctly classify known splicing events, the main purpose of the study was to describe splicing on a genome-wide scale and does not allow for detailed analysis of individual transcript structures and individual splicing events. Furthermore, the current analyses did not intend to provide insights into the functional consequences of individual splicing events, that is, whether the predicted splicing events yield functionally different protein isoforms. There is increasing evidence that a great amount of expressed transcripts result from splicing noise [11,40,41]. This is true especially for the large number of non-specific, non-conserved, and non-abundant transcripts that are frequently subjected to degradation by the regulatory mechanism nonsense-mediated decay [20].

## Conclusions

This study provides evidence for a high degree of variation among CRC samples with regards to amounts of differential exon usage. Based on this, we suggest TIN as a characteristic of CRC, which can be further dissected into the oTIN and sTIN subclasses. The oTIN subtype, reflecting sample-wise total relative amounts of deviating exon usage, is negatively correlated to the expression level of the majority of splicing factor encoding genes. Furthermore, analyses of corresponding clinical data demonstrate that TIN is associated with poor patient survival.

## Abbreviations

CEL: cell intensity; CI: confidence interval; CIMP: CpG island methylator phenotype; CIN: chromosomal instability; CRC: colorectal cancer; FIRMA: Finding Isoforms using Robust Multichip Analysis; HR: hazard ratio; MSI: microsatellite instability; oTIN: overall transcriptome instability; RMA: robust multi-array average; sTIN: skewed transcriptome instability; TIN: transcriptome instability.

## Authors' contributions

AS and THÅ conducted the exon microarray experiments and data analyses, and AS drafted the manuscript. AN and TOR collected and contributed the clinical specimens and data. RAL and RIS conceived and directed the project. All authors participated in drafting the manuscript and have read and approved the final manuscript.

## Competing interests

The authors declare that they have no competing interests.

## Supplementary Material

Additional file 1 **Supplementary methods and results**. Click here for file

Additional file 2 **Supplementary Figures 1 to 4, and Supplementary Tables 1 to 5**. Click here for file
