# Supplementary material for: Transcriptome instability in colorectal cancer identified by exon microarray analyses: Associations with splicing factor expression levels and patient survival
Source: Genome Med. 2011 May 27;3(5):32. doi: 10.1186/gm248 (PMC3219073; doi:10.1186/gm248)
Supplement: Additional file 1 — Supplementary methods and results. [file gm248-S1.PDF]

Additional file 1

Supplementary Methods

R code for running the FIRMA algorithm

Preprocessing and alternative splicing detection on CEL-files storing data from the Affymetrix GeneChip Human Exon 1.0 ST Array were done using the FIRMA algorithm [29] implemented with an R code in the aroma.affymetrix Bioconductor package [42]. R codes were retrieved from the aroma.affymetrix web page [43]. A custom made chip definition file for the core set of exons (HuEx-1_0-st-v2,coreR3,A20071112,EP.cdf) was downloaded from aroma.affymetrix [31].

The folder structures for reading input data were as follows:

CEL-files: aroma.affymetrix\rawData\a\HuEx-1_0-st-v2; a = subfolder name

CDF-files: aroma.affymetrix\annotationData\chipTypes\HuEx-1_0-st-v2

Commands used to set up the working directory and load the Bioconductor library:

```
setwd("aroma.affymetrix")
library(aroma.affymetrix)
verbose<-Arguments$getVerbose(-8)
timestampOn(verbose)
```

Command used to define the Affymetrix chip type:

```
chiptype<-"HuEx-1_0-st-v2"
```

Commands used to set up the custom made CDF-file:

```
cdf<-AffymetrixCdfFile$fromChipType("HuEx-1_0-st-v2,coreR3,A20071112, EP ")
print(cdf)
```

Commands used to set up the CEL-files to be analyzed with the above CDF-files:

```
cs<-AffymetrixCelSet$fromName("a", cdf=cdf)
print(cs)
```

Commands used to set up and run the background correction method for the above set of CEL-files:

```
bc<-RmaBackgroundCorrection(cs)
csBC<-process(bc, verbose=verbose)
```

Commands used to set up and run quantile normalization for the background corrected files:

```
qn<-QuantileNormalization(csBC, typesToUpdate="pm")
print(qn)
csN<-process(qn, verbose=verbose)
print(csN)
```

Commands used to set up and fit a probe level model for the normalized data to do transcript level summarizations:

```
plmTr<-ExonRmaPlm(csN, mergeGroups=TRUE)
print(plmTr)
fit(plmTr, verbose=verbose)
```

Commands used to set up and run the FIRMA algorithm on the pre-processed data:

```
firma<-FirmaModel(plmTr)
fit(firma, verbose=verbose)
fs<-getFirmaScores(firma)
```

Command used to extract the FIRMA scores:

```
firmaScore<-extractDataFrame(fs, units=NULL, addNames=TRUE)
```

Command for writing the data frame with FIRMA scores to a tab-delimited text file:

```
write.table(firmaScore, “firmaScore.txt”, sep=”\t”)
```

The FIRMA scores were then log-2 transformed, and the lower and upper 1st percentiles of FIRMA scores were used as thresholds for aberrantly spliced probe sets. The amounts of probe sets exceeding these thresholds were then summarized per sample.

Comparison of deviating exon usage patterns between cancer and normal tissue samples

To investigate whether the large sample-to-sample variation in deviating exon usage amounts is cancer specific, the relative amounts of deviation in exon usage in thirteen paired CRC and normal mucosa samples from the validation series were compared. The cancer and normal samples were expected to be highly variable in terms of alternative splicing patterns. Since the FIRMA algorithm uses a comparative approach to score exons as differentially spliced relative to the other samples included in the analysis, comparing two such sample groups are expected to introduce biases. To avoid this, each sample pair was individually analyzed using the FIRMA algorithm together with all the normal samples. With this approach, the difference in each sample pair in terms of exon usage deviation was normalized against the background of alternative splicing occurring in normal tissue. The upper and lower 1st percentile values of FIRMA-scores used as thresholds for denoting exons as aberrantly spliced were obtained by analyzing all the thirteen normal samples only (log2-transformed lower and upper 1st percentiles were -2.3 and 1.9,

respectively). The results for each of the sample pairs from the thirteen individual analyses were then compared directly.

Construction of random gene sets with 280 genes

For comparison to the set of 280 splicing factor genes, one hundred random gene sets of equal sizes were constructed by random sampling using the following command in the R statistical software (version 2.11.1, R foundation, Vienna, Austria):

> expr[sample(c(1:22011), 280, replace=F),], expr = genome-wide expression matrix with 22,011 transcript clusters for the CRC test series

This command randomly selected 280 genes (without replacement) from the genome-wide expression matrix for the CRC test series. One hundred iterations of random sampling were conducted to construct one hundred random gene sets of size 280. For each random gene set, Pearson correlation coefficients were calculated between gene expression levels and the total relative amounts of deviating exon usage per sample, using the Bioconductor package WGCNA [44] and the following command:

> correlation<-corAndPvalue(r, oTIN, alternative="two.sided"), r=random gene expression matrix with genes in columns and samples in rows, oTIN=vector with sample-wise deviating exon usage amounts

The gene sets were individually sorted and ranked by their Pearson correlation coefficients, and the median coefficients for the appropriate rankings across the hundred gene sets were chosen to represent the median random gene set. The corresponding significance levels for the correlation coefficients were similarly calculated as the median across the gene sets.

Permutations of total relative amounts of deviating exon usage across the test series

The total relative amounts of deviating exon usage across the 83 CRC samples were permuted to allow for comparison of correlation patterns to splicing factor expression levels between these permutations and the observed amounts of deviating exon usage. Permutation was done using the following command in the R statistical software:

sample(a), where a = a vector with the sample-wise amounts of deviating exon usage

This permutation was repeated one thousand times. Pearson correlation to splicing factor expression levels were then calculated for each permutation, as described above.

The median correlation coefficient across all splicing factor genes in each permutation was calculated and compared with the corresponding median correlation for the observed deviating exon usage amounts.

Supplementary Results

Validation of TIN in an independent series of stage II and III colorectal cancer

Transcriptome instability was tested in an independent validation series of 77 stage II and III CRCs. In the same manner as for the test sample series, exon microarray CEL-files from the validation series were analyzed for alternative splicing using the FIRMA algorithm. Sample-wise amounts of deviating exon usage were summarized based on the lower and upper 1st percentile thresholds of FIRMA scores. The log-2 transformed FIRMA scores in the validation series were normally distributed and ranged from -22.4 to 21.6 (median 0.0, variance 0.7). The log-2 transformed lower and upper 1st percentiles of FIRMA scores were -2.4 and 2.1, respectively, and were used as scoring thresholds. The amounts of deviating exon skipping and inclusion events per sample ranged from 872 to 9,610 and from 355 to 11,464, respectively. As for the test sample series, the amounts of deviating exon skipping, inclusion and exon usage, the latter being the sum of the two former, were reported relative to the respective averages across the series (Figure 5A). Tumors with deviating amounts from the average among the cancer samples were designated with sTIN and oTIN according to the thresholds as determined in the test series, *i.e.* tumors showing preferential exon skipping or inclusion (difference in relative amounts of deviating skipping and inclusion > ±0.7, n = 24) were considered to have the sTIN subtype, and tumors with total amounts of deviating exon usage larger than the common range (total relative amounts of deviating exon usage > ±1.0, n = 30) were considered to have the oTIN subtype. In this series, the percentages of samples with the sTIN and oTIN subtypes were 31 and 39, respectively, both larger than the corresponding percentages in the test series (14 and 17). Also, the number of samples with overlapping characteristics, *i.e.* designated with both sTIN and oTIN, was larger in

the validation sample series (n = 11 (13 %) compared to n = 2 (2 %) in the test series). However, there was no significant overlap between the two TIN subtypes in either of the two sample series (by Fisher's exact test). As for the test series, there were no significant associations between either of the two TIN subtypes and tumor stage, MSI-status, patient age and gender, or tumor location (as measured by multinomial logistic regression comparing the relative amounts of deviating exon usage, *i.e.* the differences in relative amounts of deviating skipping and inclusion for sTIN, and the total relative amounts of deviating exon usage for oTIN).
