# Supplementary material for: Transcriptome instability in colorectal cancer identified by exon microarray analyses: Associations with splicing factor expression levels and patient survival
Source: Genome Med. 2011 May 27;3(5):32. doi: 10.1186/gm248 (PMC3219073; doi:10.1186/gm248)
Supplement: Additional file 2 — Supplementary Figures 1 to 4, and Supplementary Tables 1 to 5. [file gm248-S2.PDF]

Additional file 2

Supplementary Figures

Supplementary Figure 1. Distribution plot of all alternative splicing (FIRMA) scores in the test series of colorectal cancers. Numbers of log-2 transformed FIRMA scores for all 284,258 exons in 83 CRC samples in the test series are included. The FIRMA scores ranged from -24.3 to 15.0 with median value 0.0 and variance 0.6. The lower and upper 1st percentiles were used as threshold values to score exons with deviating skipping or inclusion (-2.2 and 1.9 respectively, marked in red). To investigate whether different thresholds for scoring exons with deviating skipping and inclusion would alter the distribution of sample-wise amounts of deviating exon usage, we tested the upper and lower 10th and 0.1st percentiles as well. The results from the 1st percentile thresholds had Pearson correlations ≥ 0.96 with the results from both alternative thresholds, for both deviating exon skipping and inclusion events ($P < 0.001$). Hence, the sample-wise distribution of deviating exon usage amounts remained largely unaltered.

Supplementary Figure 2. Comparison of deviating exon usage in paired colorectal cancer and normal colonic mucosa samples. a) There was significantly more deviating exon usage in the cancer samples compared to their normal mucosa counterparts. Eleven out of thirteen sample pairs had higher amounts in the cancer. The mean difference in total relative amounts of deviating exon usage was 1.2 (log2-scale; $P = 0.003$, by paired samples t-test for equality of means). This was true also for the individual events deviating exon skipping and inclusion (mean difference 1.2 with $P = 0.001$, and mean difference 1.2 with $P = 0.006$, respectively). b) The mean paired differences in alternative splicing (FIRMA) scores between thirteen paired cancer

and normal samples for thirteen exons known to be alternatively spliced between CRC and normal colonic mucosa (log2-scale). All six exons known to be primarily expressed in normal mucosa (blue bars), had higher mean paired alternative splicing scores in the normal samples, Of the seven exons known to be primarily expressed in CRC (red bars), five had higher mean alternative splicing scores in the CRC samples.

Supplementary Figure 3. Correlation to splicing factor expression levels for permutations of the total relative amounts of deviating exon usage across the test series. a) The total relative amounts of deviating exon usage across the 83 CRC samples were correlated to splicing factor (n = 280) expression levels (median Pearson correlation coefficient, r = -0.23). This value was stronger than all median Pearson correlations across 1000 permutations (hence, P < 0.001), which ranged from -0.17 to 0.18 (median = 0). b) For the observed amounts of deviating exon usage, there was 2.5 times more negatively than positively correlated splicing factor genes (log2-scale), and only 10 out of the 1,000 permuted values showed a stronger tendency towards negative correlation.

Supplementary Figure 4. Validation of the associations between splicing factor expression levels and the overall TIN subtype. a) Pearson correlation coefficients for the 133 splicing factors whose expression levels were significantly correlated to the sample-wise total relative amounts of deviating exon usage in the validation series (P < 0.05). There were 129 negatively and 4 positively correlated splicing factors. b) Unsupervised hierarchical clustering analysis of all the 77 samples based on the expression levels of all 280 splicing factor genes. As for the test sample series, the validation series was also separated into clusters of samples with

predominantly low (blue boxes) or high (red boxes) amounts of deviating exon usage. c) Unsupervised hierarchical clustering analysis of the 30 samples designated with the oTIN subtype based on the expression levels of all 280 splicing factors. As for the test sample series, also the validation samples with low (n = 21) and high (n = 9) amounts of deviating exon usage were almost completely separated into two different clusters. Both clusters were created using Euclidean distance metrics and complete linkage.

Supplementary Figure 1

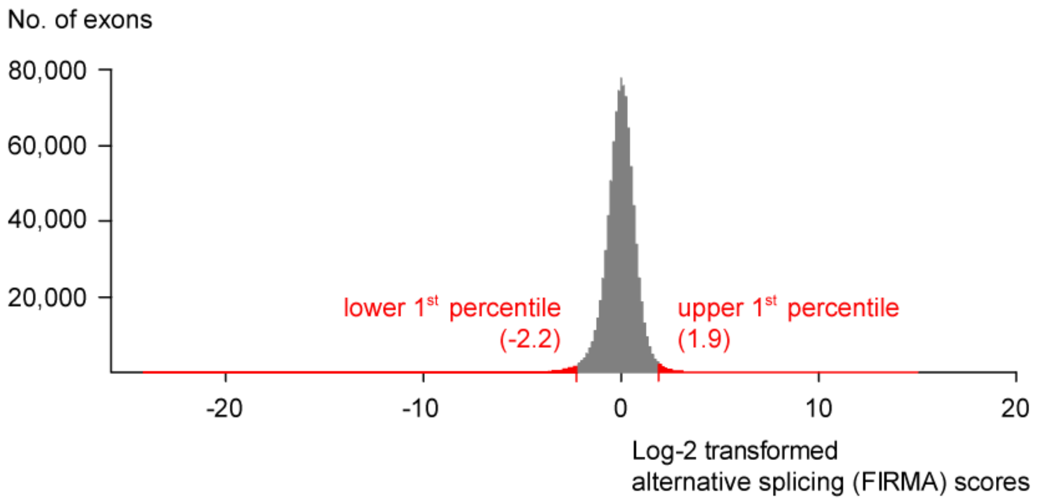

Supplementary Figure 2

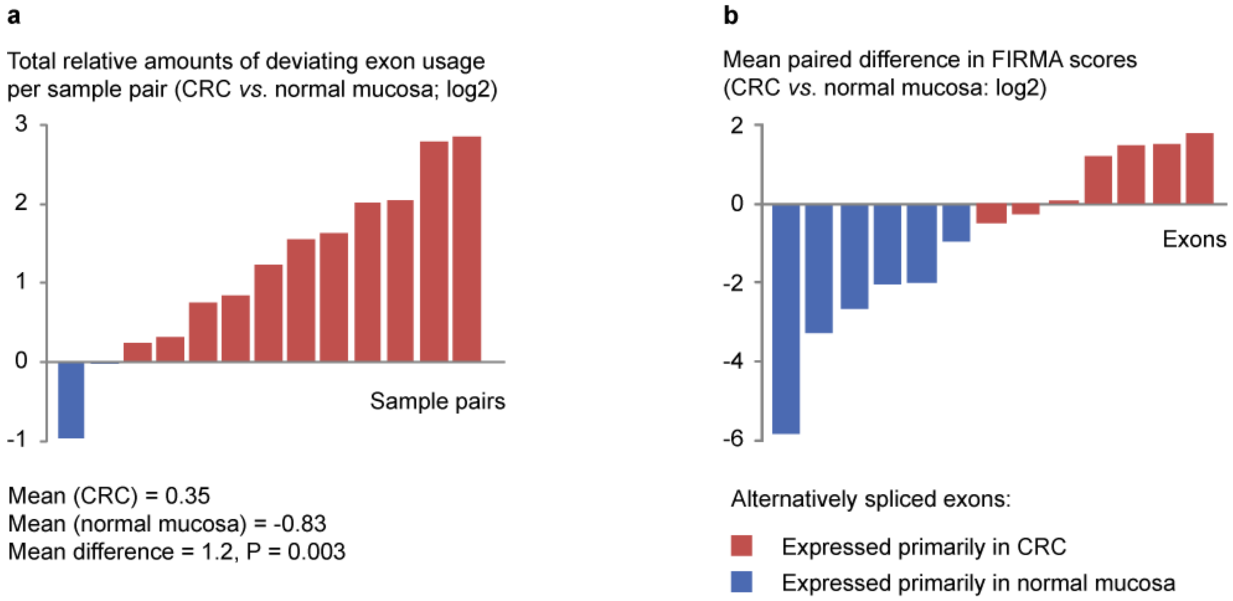

Supplementary Figure 3

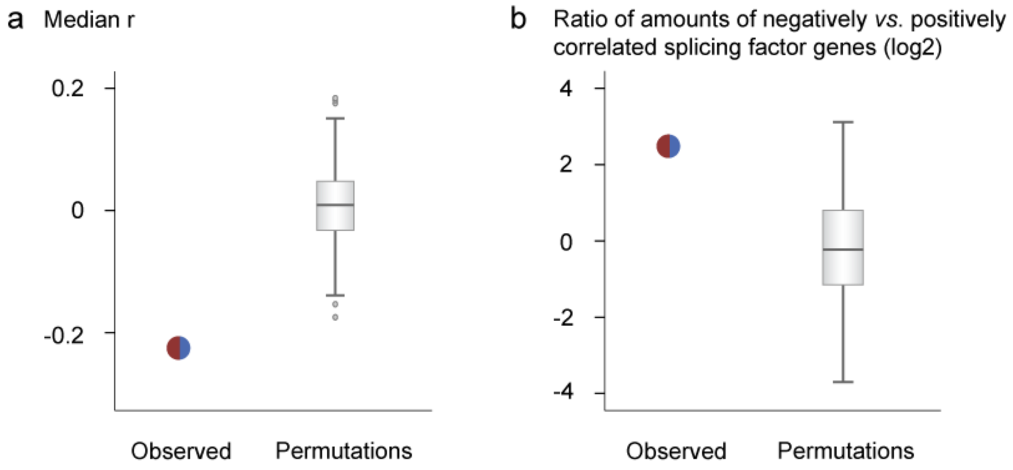

Supplementary Figure 4

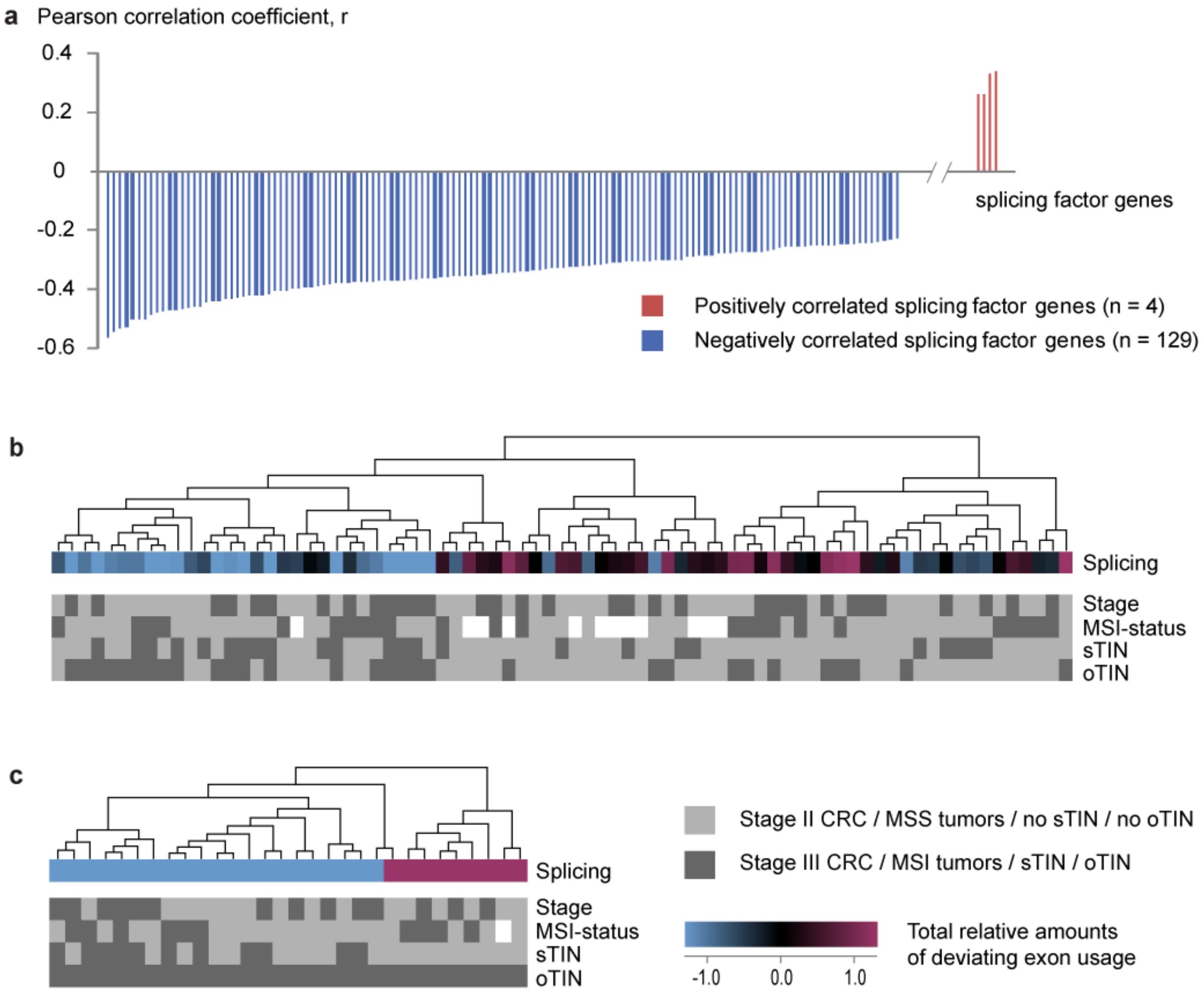

Supplementary Table 1. Sources for the gene list of 280 splicing factors

Affymetrix Transcript Cluster ID	HGNC symbol*	Gene Ontology†		Swiss-Prot		Gene Cards
		GO:0000398	GO:0005681	Splicing	Spliceosome	
2320048	*TARDBP*			x		
2325526	*SRRM1*	x		x	x	
2327259	*PPP1R8*			x	x	
2331602	*PPIE*			x	x	
2332711	*PPIH*		x		x	
2336383	*PRPF38A*			x	x	
2341565	*SFRS11*	x		x	x	
2348060	*PTBP2*			x	x	
2350287	*PRPF38B*			x		
2351940	*DDX20*	x		x	x	
2353773	*TTF2*			x	x	
2356181	*RBM8A*	x		x	x	
2358171	*PRPF3*	x	x	x	x	
2358743	*SCNM1*			x		
2370991	*DHX9*	x		x	x	
2401275	*HNRNPR*	x		x		
2402068	*SYF2*			x	x	
2403470	*DNAJC8*	x		x	x	
2403740	*SFRS4*	x		x	x	
2404377	*SNRNP40*	x	x	x	x	
2406064	*SFPQ*			x		

2406722	*LSM10*			x	x	
2407439	*SF3A3*	x	x	x	x	
2413008	*RPS13*			x		
2413180	*MAGOH*	x		x		
2418000	*ZRANB2*			x		
2421782	*RBMX*	x		x	x	
2422227	*GEMIN8*	x		x		
2427930	*WDR77*	x				
2434159	*SF3B4*	x	x	x	x	
2435149	*TNRC4*	x		x		
2448073	*IVNS1ABP*		x	x	x	
2464499	*HNRNPU*	x		x		
2468920	*CPSF3*	x		x	x	
2470654	*DDX1*	x			x	
2477980	*GEMIN6*	x		x	x	
2487527	*SNRNP27*			x		
2487639	*PCBP1*	x		x	x	
2491702	*USP39*	x			x	
2514441	*PPIG*				x	
2544179	*SF3B14*	x		x	x	
2545811	*PPM1G*					x
2548970	*SFRS7*	x		x	x	
2562115	*LSM3*			x		
2565262	*ASCC3L1*	x	x		x	
2575134	*POLR2D*	x		x	x	
2581548	*PRPF40A*			x	x	
2587747	*CIR*			x	x	

2593670	*SF3B1*	x	x	x	x	
2594497	*CLK1*					x
2594535	*PPIL3*				x	
2598099	*BARD1*					x
2611122	*TSEN2*			x		
2622469	*RBM5*	x	x	x	x	
2623154	*RBM15B*	x		x	x	
2629693	*PPP4R2*			x	x	
2649532	*RSRC1*					
2655773	*POLR2H*	x		x	x	
2694617	*ISY1*			x	x	
2697331	*DBR1*			x	x	
2709062	*SFRS10*	x		x	x	
2713074	*NCBP2*	x		x		
2728448	*POLR2B*	x		x		
2746269	*LSM6*			x	x	
2756029	*FRG1*			x	x	
2763805	*DHX15*		x	x	x	
2772017	*YTHDC1*			x		
2775463	*HNRNPD*	x		x	x	
2790570	*PLRG1*				x	
2809885	*SKIV2L2*			x	x	
2812273	*PPWD1*			x	x	
2812539	*SFRS12*			x	x	
2824315	*ZRSR2*		x	x	x	
2829488	*DDX46*			x	x	
2834093	*TCERG1*					x

2837499	*LSM11*			x	x	
2859494	*SFRS12IP1*			x		
2877141	*HNRNPA0*	x		x	x	
2881521	*RBM22*			x		
2882897	*GEMIN5*	x		x		
2884658	*SLU7*	x	x	x	x	
2890148	*HNRNPH1*	x		x	x	
2892738	*PRPF4B*			x	x	
2893847	*SNRNP48*		x	x	x	
2904248	*SNRPC*	x	x	x	x	
2905118	*SFRS3*	x		x	x	
2908572	*CDC5L*			x		
2921086	*CDC40*	x	x		x	
2934089	*WTAP*			x		
2935475	*QKI*			x	x	
2944068	*DEK*					x
2948485	*DHX16*			x	x	
2949038	*BAT1*	x		x	x	
2949431	*LSM2*	x		x	x	
2951674	*SRPK1*			x		
2952065	*PPIL1*				x	
2963407	*SYNCRIP*			x	x	
2964052	*SRRP35*	x		x	x	
2966253	*SFRS18*					x
2977690	*SF3B5*	x		x	x	
3020804	*LSM8*	x		x		
3041519	*TRA2A*	x		x	x	

3041550	*TRA2A*					x
3042421	*HNRNPA2B1*	x	x	x	x	
3044753	*LSM5*			x		
3065015	*POLR2J*	x		x	x	
3066297	*SRPK2*	x			x	
3071636	*RBM28*			x	x	
3072014	*TNPO3*					x
3098935	*TGS1*	x				
3109191	*POLR2K*	x		x	x	
3117384	*KHDRBS3*					x
3131844	*LSM1*			x	x	
3145020	*KIAA1429*			x		
3157817	*PUF60*			x		
3158516	*CPSF1*	x			x	
3168508	*MELK*					x
3181302	*NCBP1*	x		x		
3185558	*PRPF4*	x	x	x		
3199790	*PSIP1*					x
3205033	*YBX1*	x		x	x	
3212294	*HNRNPK*	x		x	x	
3233547	*RBM17*			x	x	
3234760	*CUGBP2*					x
3235932	*PRPF18*		x	x	x	
3240340	*WAC*				x	
3249738	*HNRNPH3*	x		x		
3249788	*CCAR1*	x		x	x	
3277662	*UPF2*					x

3286286	*HNRNPF*	x		x	x	
3306516	*SMNDC1*			x	x	
3311775	*DHX32*					x
3331392	*CLP1*	x		x	x	
3333622	*POLR2G*	x		x	x	
3335774	*SART1*			x	x	
3335907	*SF3B2*	x	x	x	x	
3336422	*RBM4*			x		
3342525	*PCF11*			x	x	
3358492	*POLR2L*	x		x	x	
3368304	*WT1*					x
3368520	*CSTF3*	x		x	x	
3372253	*CUGBP1*	x		x		
3375049	*PRPF19*			x		
3375340	*CPSF7*	x		x	x	
3376779	*TRPT1*			x		
3377044	*SF1*	x	x		x	
3378411	*RBM4B*			x		
3382948	*CLNS1A*	x		x	x	
3387171	*CWC15*	x		x		
3406421	*STRAP*			x	x	
3414104	*PRPF40B*			x	x	
3416036	*PCBP2*	x		x	x	
3416483	*HNRNPA1*	x	x	x	x	
3427014	*SNRPF*	x	x		x	
3435681	*ARL6IP4*			x		
3438417	*SFRS8*	x				

3439268	*NHP2L1*	x		x		
3444195	*MAGOHB*			x		
3445670	*WBP11*			x		
3451318	*ZCRB1*		x	x	x	
3452145	*SFRS2IP*	x			x	
3453319	*DDX23*	x	x		x	
3470253	*SART3*					
3474502	*SFRS9*	x				x
3475679	*ZCCHC8*			x	x	
3482112	*PABPC1*			x	x	
3486807	*WBP4*			x	x	
3494502	*DHX9*					
3503224	*UPF3A*					x
3529082	*PABPN1*	x		x		x
3533397	*SIP1*	x	x	x	x	
3533435	*PNN*			x	x	
3534201	*PRPF39*			x		
3536706	*LGALS3*					
3542207	*SFRS5*	x				x
3543411	*RBM25*	x		x	x	
3548788	*CPSF2*	x		x	x	
3550392	*PAPOLA*	x			x	
3551935	*WDR25*					
3556888	*RBM23*					x
3558745	*NOVA1*			x		x
3573261	*SNW1*	x	x	x	x	
3584443	*SNRPN*	x	x	x	x	

3617757	*AQR*			x	x	
3632107	*BRUNOL6*			x	x	
3633522	*SNUPN*					
3642162	*SNRPA1*	x	x	x	x	x
3642572	*SNRNP25*		x	x	x	
3645253	*SRRM2*			x	x	
3646613	*A2BP1*			x		
3656904	*FUS*	x		x		
3662750	*POLR2C*	x		x	x	
3665262	*NOL3*			x	x	
3665949	*PSKH1*					
3666189	*PRMT7*	x		x		x
3667281	*SF3B3*	x	x	x	x	
3667902	*DHX38*	x		x	x	
3674960	*LUC7L*					
3676669	*RNPS1*	x		x	x	x
3687698	*CD2BP2*	x		x	x	
3692895	*NUDT21*	x		x		
3698055	*TXNL4B*			x		
3708704	*POLR2A*	x		x		
3722554	*DHX8*		x	x	x	
3726772	*CROP*			x	x	
3730899	*DDX42*					
3734865	*TSEN54*			x		x
3739812	*GEMIN4*	x		x	x	
3740479	*PRPF8*	x	x	x	x	
3742708	*C1QBP*					

3759356	*EFTUD2*	x	x	x	x	x
3764103	*SFRS1*	x			x	
3766893	*DDX5*			x	x	
3771800	*SFRS2*	x		x	x	
3773312	*EIF4A3*			x	x	
3774331	*THOC4*	x		x	x	
3781082	*SNRPD1*	x			x	
3795680	*THOC1*			x		
3804452	*BRUNOL4*	x		x		
3814734	*TXNL4A*	x	x	x	x	
3815165	*PTBP1*	x		x	x	
3816333	*SF3A2*	x	x		x	
3819543	*HNRNPM*	x	x	x	x	
3820161	*UBL5*					
3820342	*PPAN*			x	x	x
3821805	*MORG1*	x	x	x		
3825383	*UPF1*					
3833757	*SNRPA*	x	x	x	x	x
3834089	*HNRNPUL1*	x		x	x	
3835983	*SFRS16*			x		
3836044	*GEMIN7*	x		x	x	
3838185	*SNRNP70*	x	x	x	x	
3838757	*SCAF1*			x		
3841231	*PRPF31*	x			x	
3841310	*TSEN34*			x		
3842345	*U2AF2*	x	x	x	x	
3844952	*POLR2E*	x		x	x	

3845868	*LSM7*	x		x	x	
3847814	*KHSRP*			x		
3848437	*XAB2*			x	x	
3852691	*DDX39*	x		x	x	
3854892	*LSM4*		x	x	x	
3855410	*SFRS14*			x		
3855660	*SF4*	x		x	x	
3859915	*U2AF1L4*			x	x	
3860277	*POLR2I*	x		x	x	
3861617	*HNRNPL*	x		x	x	
3865568	*SNRPD2*	x	x		x	
3877776	*SNRPB2*	x	x	x	x	
3882720	*RALY*			x	x	
3884922	*DHX35*			x	x	
3886050	*SFRS6*	x				
3890154	*CSTF1*	x		x	x	
3893849	*PRPF6*	x	x	x	x	
3894995	*SNRPB*	x	x	x	x	
3899954	*CRNKL1*	x	x	x	x	
3904226	*RBM39*			x		
3918696	*SON*					
3928866	*SFRS15*					x
3933999	*U2AF1*	x	x	x	x	x
3940185	*SNRPD3*	x	x		x	
3945133	*POLR2F*	x		x	x	
3952508	*DGCR14*			x	x	
3955875	*TFIP11*		x	x	x	

3956984	*ZMAT5*		x	x	x	
3957260	*SF3A1*	x	x		x	
3959203	*RBM9*			x		
3961955	*PHF5A*	x	x	x		
3962054	*NHP2L1*					
3969946	*ZRSR2*		x	x	x	x
3975987	*RBM10*			x		
3980887	*NONO*			x		
3984536	*CSTF2*	x		x	x	
3984779	*HNRNPH2*	x		x	x	
4000269	*GEMIN8*	x		x		
4009238	*SMC1A*	x		x	x	
4019570	*UPF3B*	x		x	x	
4020444	*THOC2*			x		
4045780	*FUSIP1*	x		x		
4052378	*SNRNP35*			x	x	

[*]HGNC: Human genome nomenclature committee (http://www.genenames.org). [†]GO:0000398 denotes "nuclear mRNA splicing, via spliceosome", GO:00005681 denotes "spliceosomal complex".

Supplementary Table 2. Alternative splicing (FIRMA) scores for known alternative splicing events between CRC and normal colonic mucosa

Alternatively spliced gene	Affymetrix probe set targeting alternatively spliced exon(s)		Mean difference in paired FIRMA scores*	Source
	Expressed in CRC	Expressed in normal mucosa		
SLC39A14 (Ex4A and 4B)	-	3089375	-5.8	Thorsen *et al.*
	3089381 and 3089382	-	1.5	
ACTN1 (Ex19a and 19b†)	3569830	-	1.2	Gardina *et al.* ‡
ATP2B4 (Ex21)	-	2375764, 2375765 and 2375766	-3.3	
VCL (Ex19)	-	3252128 and 3252129	-2.7	
CALD1 (extended Ex5 and Ex6)	-	3025632 and 3025638	-2.0	
SLC3A2 (Ex2, 3 and 4)	3333716 and 3333717	-	0.1	
COL6A3 (Ex3 and 4)	2605390, 2605391, 2605395 and 2605396	-	-0.5	
COL6A3 (Ex6)	2605386	-	1.5	
CTTN (Ex11)	3338589	-	1.8	
FN1 (Ex25)	-	2598321	-0.9	
TPM1 (Ex7 and 8)	3597384	-	-0.2	
	-	3597388	-2.0	

*CRC vs. normal mucosa; n = 13 pairs; log2

†Probe set targeting exon 19b (3569827) was not assigned a FIRMA score using the custom made annotation file

‡Splicing events marked with confidence level "good"

Supplementary Table 3. Genes correlated to relative amounts of aberrant alternative exon usage within the splicing factor gene set and one hundred random gene sets of equal size in the test sample series

	Splicing factor gene set	Median of 100 random gene sets	*P*-value
n (significantly correlated genes)	151	102	< 0.0001*
n (negative significant correlation; positive significant correlation)	144; 7	78; 24	< 0.0001†
Mean r‡, all 282 genes	-0.21	-0.07	< 0.0001§
Mean r‡, significantly correlated genes	-0.33	-0.19	< 0.0001§
Mean |r|**, all 282 genes	0.24	0.18	<0 .0001§
Mean |r|**, significantly correlated genes	0.36	0.32	0.002§

* *P*-value by Fisher's exact test comparing the amounts of significantly correlated genes to non-significantly correlated genes in the two gene sets

† *P*-value by Fisher's exact test comparing the amounts of significant negatively correlated genes to significant positively correlated genes in the two gene sets

‡ r = Pearson correlation coefficient

§ *P*-value by independent samples t-test for equality of means in the two gene sets

**|r| = absolute value of the Pearson correlation coefficient

Supplementary Table 4. Ten years disease specific survival rates for groups of patients in the test sample series stratified by different thresholds of the skewed TIN (sTIN) phenotype

Threshold[*]	Disease specific survival (%)[†]		*P*-value[‡]	n		n (events)	
	Within threshold	Outside threshold		Within threshold	Outside threshold	Within threshold	Outside threshold
+/-0.9	54	14	0.008	76	7	35	6
+0.9	51	0	0.12	82	1	40	1
-0.9	53	17	0.03	77	6	36	5
+/-0.8	55	20	0.02	73	10	33	8
+0.8	51	50	0.93	81	2	40	1
-0.8	55	13	.008	75	8	34	7
+/-0.7	56	17	0.001	71	12	31	10
+0.7	51	33	0.31	80	3	39	2
-0.7	55	11	.001	74	9	33	8
+/-0.6	55	33	0.04	65	18	29	12
+0.6	52	33	0.18	77	6	37	4
-0.6	54	33	0.13	71	12	33	8

[*]threshold = (relative amounts of aberrant exon inclusion) - (relative amounts of aberrant exon exclusion) [†] Survival rates from Kaplan Meier survival analysis. Events: dead from disease (n = 43), censored: survival throughout follow-up period (n = 44), ignored: recurrences [‡]Significance level from log rank test for equality of survival distributions

Supplementary Table 5. Disease specific survival analyses for patients in the two independent sample series divided into groups by the TIN phenotypes

A. Test series

Phenotype	Ten years disease specific survival (%)		P-value*	n		n (events)	
	Phenotype	No phenotype		Phenotype	No phenotype	Phenotype	No phenotype
sTIN†	17	56	0 .001	12	71	10	31
oTIN‡	43	52	0.43	14	69	8	33
Either sTIN or oTIN	33	58	0.02	24	59	16	25
Both sTIN and oTIN	0	52	0.02	2	81	2	39

B. Validation series

Phenotype	Five years disease specific survival (%)		P-value*	n		n (events)	
	Phenotype	No phenotype		Phenotype	No phenotype	Phenotype	No phenotype
sTIN†	80	88	0.57	24	53	4	6
oTIN‡	79	91	0.20	30	47	6	4
Either sTIN or oTIN	84	88	0.83	43	34	6	4

Both sTIN and oTIN	64	91	0.03	11	66	4	6

*Significance level from log rank test for equality of survival distributions. †sTIN: Preferential exon inclusion or skipping (difference in relative amounts of aberrant exon skipping and inclusion > ±0.7) ‡oTIN: Total relative amounts of aberrant splicing > ±1.0.
